# Use of prophylactic stoma mesh is a risk factor for developing rectus abdominis muscle atrophy

**DOI:** 10.1007/s10029-022-02580-3

**Published:** 2022-04-05

**Authors:** S. Täckström, A. Chabok, K. Smedh, M. Nikberg

**Affiliations:** 1 Department of Radiology, Västmanlands Hospital Västerås, Västerås, Sweden; 2 Department of Surgery, Västmanlands Hospital Västerås, Västerås, Sweden; 3 Centre for Clinical Research of Uppsala University, Västerås, Sweden

**Keywords:** Mesh, Parastomal hernia, Risk factors

## Abstract

**Purpose:**

The aim of this study was to evaluate the possible risk factors for developing a parastomal hernia (PSH) in a cohort of rectal cancer patients with and without the application of a retro-muscular prophylactic mesh. The evaluated risk factors included the position of the stoma in the rectus abdominis muscle (RAM), RAM atrophy and shift of abdominal wall midline structures.

**Methods:**

Rectal cancer patients treated with an abdominoperineal excision or Hartmann’s procedure between 2002 and 2015 at Västmanland Hospital, Sweden was included. Postoperative CT examinations were retrospectively reviewed regarding the presence of PSH and RAM atrophy and for measurements such as position of the stoma in the RAM.

**Results:**

116 patients were included, with a median age of 71 years. 70 patients received a prophylactic stoma mesh. 55 patients (47%) had a parastomal hernia at three-year follow-up. Rectus abdominis muscle atrophy was significantly higher in the mesh group compared with the non-mesh group (37% vs 2%; *P* = 0.04). RAM atrophy was a significant independent protective factor for developing a PSH (*P* = 0.007; OR 0.15; 95% CI 0.03–0.55).

**Conclusion:**

Placement of a prophylactic retro-muscular stoma mesh resulted in a high frequency of RAM atrophy distal to the stomal aperture and patients with such atrophy had a lower risk of developing a PSH.

## Background

Parastomal hernia (PSH) is a common complication of stomal surgery that impairs quality of life [1] and sometimes requires additional surgery [2]. The incidence of PSH increases with time and was reported to reach 30% by 3 years in studies where PSHs were evaluated using computed tomography (CT) [3, 4]. The causes of PSHs are mostly unknown, although risk factors, such as the stomal aperture area [5] and high body mass index (BMI) [2, 6], have been proposed. Several trials of prophylactic mesh placement during stomal formation have been conducted, but with varying results [7–9]. The European Hernia Society now recommends prophylactic mesh placement [10], but because of the contradictory results, the increased surgical time and the possibility of surgical trauma, it is not performed routinely in Europe [11]. Further, in the last two years, large randomized trials with prophylactic retro-muscular stoma mesh placement have reported no protective effect of a PSH [12–14], and a meta-analysis including these recent studies found no effect of prophylactic mesh on the development of a PSH [15].

Placement of a stoma either trans-rectally through the rectus abdominis muscle (RAM) or para-rectally has been a focus for research [16]. At our institution, all stomas are placed trans-rectally. The RAM is thickest in the center and often thinner both medially and laterally. This led us to the hypothesis that placement of a stoma in the RAM might be a factor in the development of PSH. It has been shown that the thickness of the RAM decreases caudal to the stoma—sometimes interpreted as atrophy—for patients with no PSH, but no significant atrophy was seen among patients with a PSH [17]. The aim of this study was to evaluate possible risk factors for developing a PSH including RAM atrophy, midline shift and position in the RAM in a cohort of patients with and without the application of prophylactic mesh.

## Methods

### Study cohort

Patients with rectal cancer treated surgically with abdominoperineal excision (APE) or Hartmann’s procedure (HP) between 2002 and 2015 with a permanent stoma were identified and selected from a prospective registry for rectal cancers at department of surgery, Västmanland Hospital, Sweden. According to the department standard follow-up procedure, all patients were subjected to clinical and radiological follow-up consultations at 1 and 3 years. The patient cohort in this study included all those who had a follow-up abdominal Computed Tomography (CT) examination at 12 months (range 6–18) and at 36 months (range 30–42) after surgery.

### Surgical procedures

Between 1996 and 2006, all permanent colostomies were created without the use of prophylactic mesh. In 2007, based on the first published randomized study on preventing a PSH [18], a decision was taken to use prophylactic stoma mesh in patients with rectal cancers operated upon with APE and HP. Two different mesh types were used during the study period: Vipro^®^ (Ethicon, Norderstedt, Germany) mesh cut to 10 × 10 cm from 2007 until 2009 and Parietex ProGrip (Medtronic, Fridley, Minnesota, U.S.) mesh cut to 15 × 9 cm from 2010 onward. The mesh was placed between the RAM and the posterior rectus sheath in a sub-lay position according to the technique described by Jänes et al. [18]. There was no specific reason for the shift to the ProGrip mesh in 2010 except that at that time we thought it to be easier and faster to place.

### Assessment of CT examinations

All CT examinations were evaluated by one certified radiologist with a special interest in PSHs, regarding hernia diagnosis, RAM atrophy assessment and measurements as described below. The measurements and other assessments of the CT examinations were performed before merging with the data from the clinical registry such as the presence of a prophylactic mesh.

### Hernia diagnosis

All CT examinations were performed with the patient in a supine position, some with and some without intravenous contrast medium. The CT examinations were classified as either *no hernia* or as *parastomal hernia* and also graded according to the Moreno–Matias scale [3].

### Midline shift

**Fig. 1:**
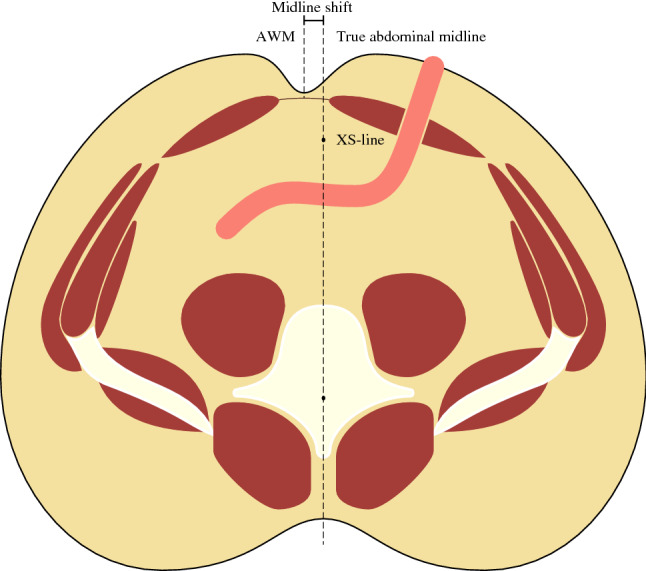
The midline shift is defined as the distance between the abdominal wall midline (AWM) and the true abdominal midline. The true abdominal midline is the plane including the straight line between the xiphoid process and the pubic symphysis (XS-line) and the center of the spinal canal

The true anatomical midline was defined as the plane containing the tip of the xiphoid process, the cranial ventral end of the pubic symphysis and the center of the spinal canal at the level of the stoma. The midline of the abdominal wall was defined as the midpoint between the left and right RAMs medial limits at the height of the stoma. A midline shift was defined as the shortest distance from the abdominal wall midline to the true anatomical midline plane, where positive and negative values represent a shift toward or away from the stoma respectively. This is illustrated in Fig. 1.

In a previous study measuring the midline shift, a virtual reality system with a dedicated headset and a virtual pointer was used [17]. We developed a specialized DICOM (Digital Imaging and Communications in Medicine) viewer for this task using the open source Visualization Toolkit [19], where the CT examinations could be viewed, the aforementioned reference points could be placed, and the midline shift was then calculated automatically.

### RAM atrophy

**Fig. 2:**
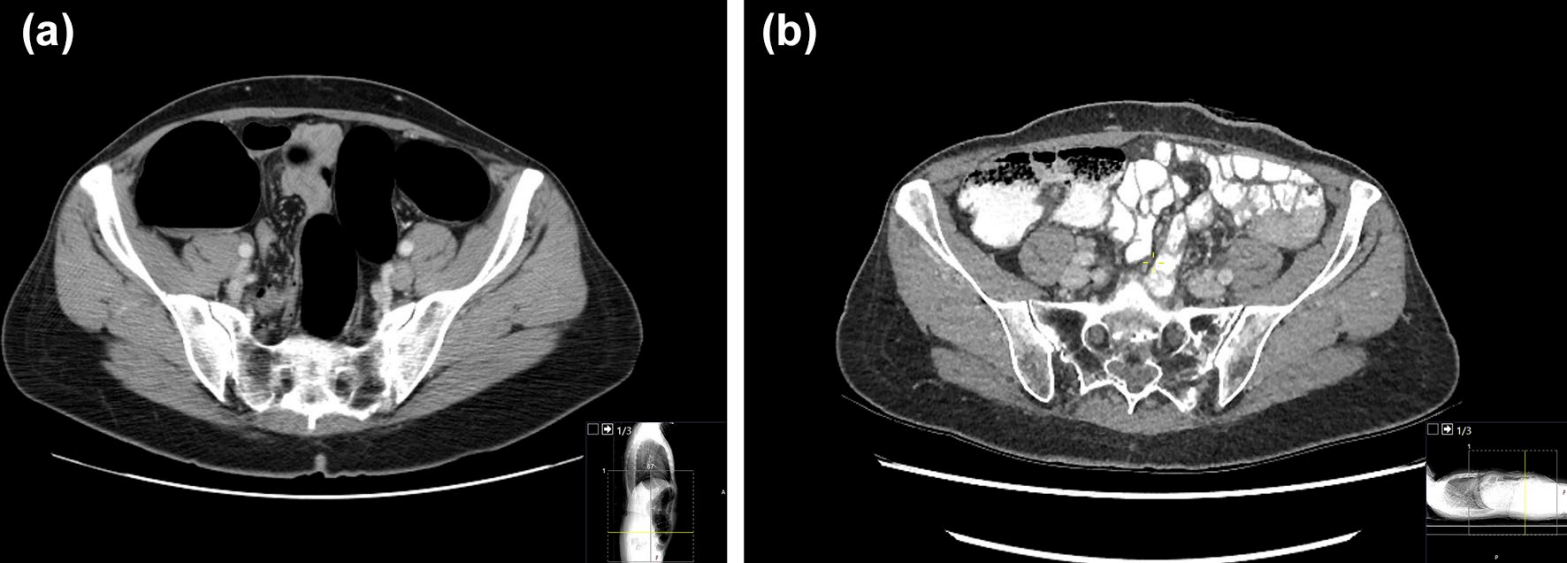
CT examinations of a patient with rectal cancer (**a**) pre- and **b** postoperative. The images are at the same level, caudal to the created stoma in the left rectus abdominis muscle (RAM). Preoperative findings of symmetric left and right RAM thickness and appearance. Postoperative marked asymmetry with normal appearance of the right RAM and a thin atrophic left RAM

The thickness of the RAM below the stoma was compared with the contralateral side and if the thickness was visually judged to be significantly thinner than that of the contralateral side, it was classified as atrophied and otherwise as equal. RAM atrophy is illustrated in Fig. 2 where a preoperative CT colonography (Fig. 2a) shows normal symmetric RAM and postoperative abdominal CT (Fig. 2b) shows an atrophic left RAM caudal to a stoma.

### Risk factors

The radiological measurements were made on the first selected CT examination for each patient (the CT after 12 months). The stoma aperture’s width (W) and height (H) were measured. The stomal aperture area was calculated by approximating the shape as a diamond (*W* × *H*/2).

The position of the stoma in the RAM was studied by measuring the distance from the medial RAM limit to the medial stoma border (*M*) and similarly from the lateral stoma border to the lateral RAM limit (*L*). Both the absolute value for M and the relative position in the RAM [100 × *M*/(*M* + L) %] were used in the analysis.

**Fig. 3:**
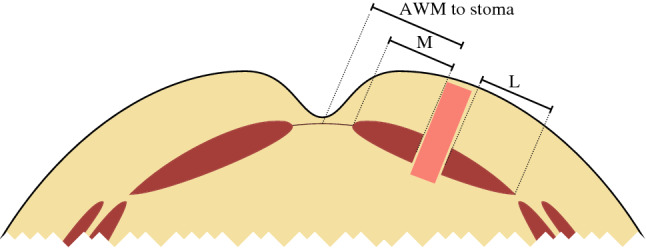
Measurements of the stomal position related to the abdominal wall midline (AWM to stoma) and in the RAM. For the latter, the length (*L*) and position (*M*) in mm and the relative position were measured as [100 × *M*/(*M* + *L*)%]

When planning the stoma placement preoperatively the medial limit of the RAM was not known, and it was easier to use the abdominal wall midline as a reference. Therefore, the distance between the abdominal wall midline and the medial stomal border was also measured. These measurements are illustrated in Fig. 3.

In the patient registry, smoking status was registered as *non-smoking*, *previously smoking* or *smoking.* The BMI value was available for every patient.

### Statistical analysis

All statistical analyses were performed with the R Statistical software version 3.5.0 [20]. *P* < 0.05 was considered statistically significant. For differences in proportions, the chi-squared test was used except for small numbers where Fisher’s exact test was used. Student’s *t* test was used for comparing mean values. For multivariable analysis, a binomial logistic regression model was used. In the multivariable analysis, the smoking values *missing*, *non-smoking* and *previous smoker* was grouped into *non-smoking.* The measurements *Stoma to abdominal wall midline* and *Stoma position in RAM* (in mm and %) are by their definition positively correlated to a high degree. From the multivariable analysis with all these variables, only the variable with the best fit was kept and the other two were discarded for the final model. Results are presented as the odds ratio (OR) and 95% confidence interval (CI).

The stomal aperture area and BMI were strongly correlated so only the variable with the best fit was kept in the multivariable analysis. When classifying stomas for PSHs, there were some borderline cases that were ambiguous. These cases were either graded as 0 or 1, while all grade 2 and 3 PSHs were judged to be definite PSHs. To validate our results further, we used the same multivariable model, but as dependent variables, we grouped them as grades 2 or 3 (definite PSH) and 0 or 1 (improbable PSH).

## Results

Of the 116 patients included, 72 (62%) were male, 70 (60%) had received placement of a prophylactic mesh and 16 (14%) were smokers. The median age was 71 years (range 44–88). The clinical and CT-measured data for patients with and without prophylactic mesh placement are presented in Table 1. RAM atrophy was significantly higher in the mesh group compared with the non-mesh group (37% vs 2%; *P* < 0.001). Only one patient without a prophylactic mesh showed RAM atrophy. In the subset of patients with a prophylactic mesh, of those who had atrophy of the RAM, 27% developed a PSH compared with 50% of those who did not, with borderline significance (*P* = 0.08). The multivariable analysis of risk factors for PSH is presented in Table 2. RAM atrophy was a significant independent protective factor for developing a PSH (*P* = 0.007; OR 0.15; 95% CI 0.03–0.55). Increasing age (*P* = 0.001; OR 1.09; 95% CI 1.04–1.16) and larger stoma aperture (*P* < 0.001; OR 1.85; 95% CI 1.45– 2.50) were also found as risk factors for developing PSH. In the final multivariable analysis defining a definite PSH as those with grades 2 and 3, in addition to age, large stomal aperture and RAM atrophy, the position of the stoma in the RAM was found to be a significant risk factor for the development of a PSH (*P* = 0.03; OR 1.89; 95% CI 1.09–3.43; Table 3).

**Table 1:** Radiological and clinical data on patients with rectal cancers and a colostomy with and without a prophylactic stoma mesh in the Västmanland region between 2002 and 2015

	No stoma mesh	Stoma mesh	*P*
Total	46	70	
Age (y)^a^	74 (48–88)	70 (44–86)	0.06
Gender			1
Male	29 (63)	43 (61)	
Smoking			0.09
No	16 (35)	37 (53)	
Past	22 (48)	21 (30)	
Yes	7 (15)	9 (13)	
Missing	1 (2)	3 (4)	
BMI (kg/m^2^)^b^	26.7 (4.3)	26.7 (3.9)	0.92
RAM atrophy			** < 0.001**
No	45 (98)	44 (63)	
Yes	1 (2)	26 (37)	
Midline shift (cm)^b^	− 0.47 (0.8)	− 0.46 (0.9)	0.94
PSH at 12 months	19 (41)	20 (29)	0.22
PSH at 36 months	26 (57)	29 (41)	0.16
Stoma position in RAM (cm)^b^	3.5 (1.0)	3.8 (1.2)	0.18
Stoma position in RAM (% of total width from medial edge)^b^	57 (17)	59 (13)	0.49
Stoma distance from abdominal wall midline (cm)^b^	5.2 (1.3)	5.5 (1.3)	0.29
Stoma aperture area (cm^2^)^b^	5.1 (2.8)	4.6 (2.7)	0.36

Values in parentheses are percentages unless otherwise specified

*P*-values < 0.05 are in bold

*BMI* body mass index, *RAM* rectus abdominis muscle, *PSH* para-stomal hernia

^a^Values are medians and (range)

^b^Values are means and (standard deviations)

**Table 2:** Binomial multivariable logistic regression of para-stomal hernia risk in patients with rectal cancers and a colostomy in the Västmanland region between 2002 and 2015

	*P*	OR	95% CI
Age	**0.001**	1.09	1.04–1.16
Gender	0.606		
Male		1.0	
Female		0.76	0.26–2.18
Smoking	0.317		
No or previous		1.0	
Yes		1.99	0.52–8.02
RAM atrophy	**0.007**		
Absent		1.0	
Present		0.15	0.03–0.55
Midline shift (cm)	0.598	1.18	0.65–2.20
Prophylactic mesh used	0.765		
No		1.0	
Yes		1.17	0.43–3.27
Stomal position in the RAM (cm)	0.056	1.56	1.00–2.50
Stomal aperture area (cm^2^)	** < 0.001**	1.85	1.45–2.50

*P*-values < 0.05 are in bold

*CI* confidence interval, *OR* odds ratio, *RAM* rectus abdominis muscle

**Table 3:** Binomial multivariable logistic regression of the risk of a grade 2 or 3 para-stomal hernia according to the Moreno–Matias scale in patients with rectal cancers and a colostomy in the Västmanland region between 2002 and 2015

	*P*	OR	95% CI
Age	**0.041**	1.07	1.01–1.14
Gender	0.752		
Male		1.0	
Female		0.82	0.22–2.85
Smoking	0.878		
No or previous		1.0	
Yes		0.88	0.154–4.22
RAM atrophy	**0.019**		
Absent		1.0	
Present		0.11	0.01–0.58
Midline shift (cm)	0.244	1.47	0.78–2.94
Prophylactic mesh used	0.473		
No		1.0	
Yes		1.52	0.49–4.96
Stoma position in RAM (cm)	**0.027**	1.89	1.09–3.43
Stoma aperture area (cm^2^)	** < 0.001**	1.85	1.44–2.50

*P*-values < 0.05 are in bold

*CI* confidence interval, *OR* odds ratio, *RAM* rectus abdominis muscle

We also analyzed the two mesh types separately. For patients with the Vipro mesh, 30% (8 of 27) had RAM atrophy and with the Parietex mesh 42% (18 of 43) had RAM atrophy, this difference was not statistically significant. The multivariable analysis with PSH development was also made with the two mesh types as separate independent variables, but neither mesh type was found to be a significant factor for PSH development (Vipro *P* = 0.61; OR 1.39; 95% CI 0.39–5.15 and Parietex *P* = 0.95; OR 1.04; 95% CI 0.34–3.27) while RAM atrophy was still a significant protective factor (*P* = 0.008; OR 0.15; 95% CI 0.03–0.57). Similar results were found when analyzing with definite PSH (grade 2 and 3) as dependent factor (data not shown).

## Discussion

This is the first study to show that prophylactic stoma mesh increases the risk for RAM atrophy below the stoma. Intercostal and the iliohypogastric nerves are positioned in the area where stomas are generally created and innervate the RAM at the level of the stoma and caudally [21]. They course between the internal oblique muscle and the transverse abdominal muscle to the lateral border of the rectus sheath. Damage to those nerves by an extension of the dissection between the RAM and the deep layer of the rectus sheath laterally for the mesh placement could explain such atrophy. Here we also found—perhaps counterintuitively—that RAM atrophy was an independent protective factor for developing a PSH. One possibility is that the atrophic RAM moves less and exerts less strain on the interface between the stoma and the abdominal wall. This could lead to less pressure on the intra-abdominal tissues in the area below the stoma and allow the stomal tissues to heal along with the surrounding abdominal wall structures. Another possibility is that both RAM atrophy and the observed reduced risk for PSH are both secondary findings of an unintended difference of surgical technique when creating the stoma with a prophylactic mesh. This might also explain why previous trials have shown diverse results regarding the effect of prophylactic meshes. An additional interesting finding was that the distance of the stoma from the medial edge of the RAM was a risk factor for developing a grade 2 or 3 PSH.

More studies are needed to confirm the findings in this retrospective study. However, they are important because common sense suggests that denervation and muscle atrophy should be avoided during surgery and that atrophy would be a risk factor for PSHs. However, these results point in the opposite direction. Correlation of the stomal aperture area with a PSH has been shown before. Some caution should be taken when interpreting such causality, as it is quite probable that when a PSH develops its content will exert a force on the stomal aperture and thereby widen it.

A high BMI has been reported as a risk factor for the development of a PSH. We found that this was strongly correlated with the stomal aperture area, which makes it difficult to assess if one of these, or both, are real risk factors. This is not surprising because pericolic fat joins the colon through the stomal aperture requiring the aperture to be constructed larger, and there is an obvious correlation between BMI and the amount of pericolic fat.

The one-year incidence of PSH (29% and 41% with and without a prophylactic mesh) in this study was at the high end among the studies reported [22] but lower than a recent prospective study from Sweden with reported PSH rate of 40% and 51% in patients with and without a prophylactic mesh [23].

In this cohort, the multivariable analyses found no significant effect of a prophylactic mesh as a separate factor, but since all but one patient with RAM atrophy was in the prophylactic mesh group, and RAM atrophy was a significant protective factor, there was an overall lower incidence (not statistically significant) of PSH in the mesh group. Several randomized studies have been made evaluating the benefit of a prophylactic mesh and therefore we believe that this retrospective study does not add much evidence regarding the effect of a prophylactic mesh. In a recent meta-analysis of randomized trials regarding prophylactic meshes [22], the studies show large variation both in the effect of prophylactic mesh as well as in the overall incidence of PSH detected both clinically and radiologically. The results of this exploratory study of factors that can help explain the large variation in PSH incidence should not alter the immediate management of patients, but we believe it would be feasible to replicate this study in future randomized trials as well as retrospectively in randomized trials that already has CT examinations of patients.

### Limitations

This was a retrospective study with inherent limitations and the results need to be confirmed in other cohorts. The optimal protocol for PSH assessment with CT is with the patient in a prone position doing a Valsalva maneuver. This is not part of the standard protocol for follow-up of patients with colorectal cancers at our institution, which is aimed at finding metastatic disease, so the true PSH formation rate is probably higher than that reported here. Ideally, measurements should be made using CT scans before a PSH develops, closer in time to the surgery. The surgical technique for creating the stoma and inserting the mesh is operator-dependent; however, only three senior surgeons were involved in mesh insertion for all the patients in this cohort, so different surgical techniques between the surgeons are unlikely to have been a decisive factor; however, minor differences in technique between patients with and without mesh placement regarding the stoma creation probably exist.

RAM atrophy is not routinely assessed or measured on CT examinations. In the planning process, we considered making measurements of the RAM instead of a visual assessment. The choice of appropriate parameter(s) like thickness, width and area of the RAM as well as whether to make them in one or more locations was not clear beforehand. We presumed that such non-validated measures could give systematic bias. We therefore chose to assess the RAM visually and only classify cases as RAM atrophy if there was unambiguous atrophy as opposed to borderline cases. This leads in our view to higher validity but possibly lowered reliability in this classification. Regarding the significant correlation between prophylactic mesh and RAM atrophy, it is important to note that the prophylactic mesh is not visible on CT and was not known to the radiologist during assessment; therefore, the risk for systematic reviewer bias influencing this correlation should be low.

## Conclusion

Placement of a prophylactic retro-muscular stoma mesh resulted in a high frequency of RAM atrophy distal to the stomal aperture and patients with such atrophy had a lower risk of developing a PSH.

## Data Availability

Not applicable.
